# Supplementary material for: The association between guidelines adherence and clinical outcomes during pregnancy in a cohort of women with cardiac co-morbidities
Source: PLoS One. 2021 Jul 23;16(7):e0255070. doi: 10.1371/journal.pone.0255070 (PMC8301645; doi:10.1371/journal.pone.0255070)
Supplement: S1 Table — (PDF) [file pone.0255070.s001.pdf]

Supplementary Materials

S1 Table: Cardiac diagnosis

ICD codes	Isolated cardiac arrhythmias
R00.0, R00.1, 144.1, 144.2, 144.30	Bradyarrhythmias and Atrioventricular (AV) heart blocks.
147.1, 147.0,	Supraventricular tachyarrhythmias (SVT), i.e. atrial tachycardia and atrioventricular nodal re-entry tachycardia (AVRNT).
148.0, 148.1, 148.4, 148.9	Atrial Fibrillation (AF), and Atrial Flutter.
147.2, !49	Ventricular tachyarrhythmias: Ventricular tachycardia and ventricular fibrillation (VT and VF).
145.6	Wolff-Parkinson-White (WPW) syndrome.
145.81, 149.8	Long QT syndrome and Brugada syndrome (BrS).
	**Cardiomyopathies (CMP)**
142, 142.9	Dilated cardiomyopathy (DCM) includes idiopathic cardiomyopathy.
125.5	Ischemic cardiomyopathy (IC).
142.2, 090.3	Hypertrophic cardiomyopathy (HCCM).
090.3,099.4, 142	Peripartum cardiomyopathy (PPCM).
142.9	Tachycardiomyopathy (TIC).
150.9, 075.4	Acute decompensated heart failure unspecified event.
142.3, 142.4	Spongiform Cardiomyopathy.
	**High-risk cardiac lesions**

Supplementary Materials

S1 Table: Cardiac diagnosis

124.9, 121.4	Acute coronary syndrome (ACS), i.e. Coronary artery disease (CAD), ischemic heart disease (IHD), acute myocardial infarction (AMI).
151.9	At least mild systemic ventricular systolic dysfunction.
D15.1	Atrial Myxoma.
Q25.1, Q23.1	Coarctation + bicuspid Aortic Valve.
Q 20.8	High-risk left-sided valve disease/ Left ventricular outflow tract (LVOT) obstruction.
Q87.4	High-risk aortopathies, i.e. Marfans Syndrome, Bicuspid aortic valve with aortic arch dilation.
Z 95.2	Mechanical heart valve.
127.0	Pulmonary hypertension.
142.8	Arrhythmogenic Right Ventricular Dysplasia (ARVD).
	**Type of Congenital heart disease (CHD)**
	**Complex CHD**
Q25.6, Q23.0	Acyanotic congenital: Aortic/pulmonary stenosis with recent acute myocardial infarction, corrected as adult: ASD closure and pulmonary valvuloplasty for pulmonary valvular stenosis.
Q25.6	Anomalies of the pulmonary artery: multiple pulmonary artery stenosis with pulmonary hypertension.
Q21.3, Q22.3	Cyanotic CHD: Ventricular septal defect (VSD) corrected later, and Tetralogy of Fallot (TOF) corrected at one year, with pulmonary valve replacement 20 years later.

Supplementary Materials

S1 Table: Cardiac diagnosis

V13.65	Cyanotic CHD: Fontan procedure for double inlet left ventricle, VSD, ASD, PDA / Pulmonary stenosis/juxtaposition of the atrial appendage, corrected at three years of age.
Q 44.7, Q 20.8	CHD corrected (*Cardio hepatic dysplasia, portal hypertension and cirrhosis, with Jejunal atresia corrected at birth)* Corrective adult surgery for Closure of ASD with a pericardial patch, RVOT, PVR tissue, for severe pulmonary stenosis.
Q21.3, 745.1	Tetralogy of Fallot (TOF) Transvascular patch, right ventricular outflow tract (RVOT) corrected at 11 months (+surgery later).
Q21.3, Q23.2, 745.1	Tetralogy of Fallot (TOF) corrected with residual mild to moderate pulmonary hypertension (PH).
Q 25.6	Pulmonary stenosis**.**
	**Non-Complex**
Q21.1	Atrial septal defect (ASD).
Q21.1, Q21.0.	ASD and VSD non-surgical correction with Amplatzer device (ventricular septal aneurysm).
Q 89.3	Dextrocardia (situs inversus).
Q 25.0	Patent ductus arteriosus (PDA).
Q 21.1	Patent Foramen Ovale (PFO) discovered during pregnancy uncorrected.
Q25.0, V13.65	Patent Foramen Ovale corrected as an adult with an Amplatzer device 20 years later.
Q25.0, 745.9, 745.5	Patent Foramen Ovale corrected as an adult when found ASD as well.
Q 89.9,745.4	Ventricular septal defect (VSD).

Supplementary Materials

S1 Table: Cardiac diagnosis

	**Valvular heart disease** (VHD)
Q23.0, 106.2, 746.3, 746.4	Aortic Stenosis (AS) and Aortic insufficiency /incompetence.
Q 89.9.	Anomalies of great veins: Atresia of the aorta with patent foramen Ovale (PFO). Right-sided aortic arch with an anomalous left subclavian artery from a large-mouthed aortic diverticulum with compression of the trachea between the diverticulum and the ascending aorta- required urgent surgery during pregnancy.
127.22, Q23.2, 105.0, 134.2, 746.5,	Mitral valve stenosis (MS) and pulmonary hypertension (PH).
R11.10, Q23.3, 746.1,127.22	Mitral regurgitation (MR) accompanied by PH and Tricuspid regurgitation.
R11.10,105.2, 108.3,	Mitral insufficiency (Congenital, i.e., MR due to Mitral valve prolapse or mitral valve incompetence with increased LVOT velocity and width of ascending/descending aorta.
134.1	Mitral Valve Prolapse (MVP).
108, 135.0	Mixed Valve disease (MVD).
101.9, 105.9, 108.0,108.8	Rheumatic heart disease with MVD, MS, and AS and left ventricular hypertrophy (LVH).
107.0, 107.2.	Tricuspid valve disease: regurgitation or stenosis.
	**Myocardial conditions with cardiac involvement**
D 68.61	Antiphospholipid syndrome.
G71.09, G71.11	Maternal Myotonic Muscular Dystrophy (MMD).

Supplementary Materials

S1 Table: Cardiac diagnosis

Q87.19	Noonan syndrome.
171.0	Type B aortic dissection
ICD codes URL: https://www.cdc.gov/, https://www.who.int/classifications/icd/icdonlineversions/en/ accessed 25th August 2020.	
