# Supplementary material for: The association between guidelines adherence and clinical outcomes during pregnancy in a cohort of women with cardiac co-morbidities
Source: PLoS One. 2021 Jul 23;16(7):e0255070. doi: 10.1371/journal.pone.0255070 (PMC8301645; doi:10.1371/journal.pone.0255070)
Supplement: S2 Table — (PDF) [file pone.0255070.s002.pdf]

S 2 Table: Neonatal diagnosis of congenital heart disease and abnormalities

Maternal cardiac status	ICD -10 code	Congenital abnormality diagnosis in neonates
Preexistent cardiac condition of uncorrected VSD	Q26.8	Diagnosed (*at birth*) with Congenital Heart Disease (CHD), multiple issues: two ventricular septal defects (VSD), confirmed on echocardiogram. Congestive cardiac failure treated with diuretics. Patent ductus arteriosus (PDA), two VSDs: large perimembronous VSD, large atrial septal defects (ASD) and hemi anomalous pulmonary venous drainage. A variant of scimitar or pulmonary venolobar syndrome. The baby admitted to Neonatal Intensive Care Unit (NICU), where diuretics treated heart failure, but transferred to a paediatric facility under the care of a paediatric cardiologist.
Acquired cardiac condition unmasked during pregnancy	Q87.4	Marfans syndrome diagnosed in both mother and baby (*at birth*); therefore, genetic counselling and appropriate adult and paediatric cardiology follow-up implemented. The baby required NICU admission.
Preexistent Marfans syndrome family genetic history	Q87.4	Marfans syndrome also diagnosed in the baby. Therefore, paediatric cardiology follow-up implemented.
Preexistent cardiac condition of severe mitral stenosis, tricuspid and aortic regurgitation,	Q87.0	Facio-Auricular-Vertebral Spectrum (Goldenhar syndrome) premature delivery with baby admission to NICU where the congenital condition was diagnosed, and ongoing paediatric follow-up was implemented.

pulmonary hypertension, underwent an elective mitral valvuloplasty antepartum.		
Preexistent cardiac condition of bicuspid aortic valve and aortic incompetence with left ventricular dysfunction	Q79. 0	Diagnosis of cyanosis and CHD (*at birth*): Rapidly cyanosed within the first few minutes of birth when found to have a significant diaphragmatic hernia on chest transferred to a paediatric facility for surgery. Required intubation and mechanical ventilation on FiO2 80 % further investigations identified pulmonary hyperplasia. Ongoing paediatric cardiology follow up
Preexistent Genetic Congenital Noonan syndrome with secondary hypertrophic cardiomyopathy	Q87.1	The baby diagnosed with genetic 'Noonan syndrome' while in Special Baby Care Unit (SBCU) post-birth. Medical genetic counselling antepartum post foetal echocardiogram and post-delivery. Long term paediatric follow-up.
Preexistent cardiac condition of congenital peripheral pulmonary stenosis and pulmonary hypertension	128.8	Baby to NICU *at birth* diagnosed with pulmonary stenosis with paediatric cardiology follow-up

Legend. Scimitar or pulmonary venolobar syndrome is a rare but well known congenital cardiovascular defect accessed 12102020 via URL:

https://www.ahajournals.org/doi/full/10.1161/circulationaha.109.931857 ICD codes via URL: https://www.who.int/classifications/icd/icdonlineversions/en/.
