# Supplementary material for: The association between guidelines adherence and clinical outcomes during pregnancy in a cohort of women with cardiac co-morbidities
Source: PLoS One. 2021 Jul 23;16(7):e0255070. doi: 10.1371/journal.pone.0255070 (PMC8301645; doi:10.1371/journal.pone.0255070)
Supplement: S3 Table — (PDF) [file pone.0255070.s003.pdf]

S 3 Table: Coding of clinical interventions, complications and outcomes for SPSS Variables for obstetric neonatal and maternal cardiac.

**SPSS variable**	**Description**
ID	STUDY ID
Gravida	Gravida
Parity	Parity
Gestational_age_admission	Gestational_age_admission
Maternal_age	Maternal age
Apgar_1min	Apgar_1min
Apgar_5min	Apgar_5min
POSTCODE	PC
SCORE	Total Adherence score
ACQUIREDGROUP	Acquired cardiac group
PREEXISTENTGROUP	Pre-existent cardiac group
**Obstetric variables that include interventions, modes of delivery and complications**	
EMERGENCYLSCSHYSTRECTOMY	Emergency lower segment caesarean section (LSCS) + Hysterectomy/Laparotomy
EMERGENCYLSCS	Emergency LSCS
DELIVERYMODELSCS	(Elective) LSCS
DELIVERYNVD	Normal vaginal delivery (NVD)

DELVERYASSISTED	Assisted vaginal delivery (AVD)
DEVIATIONDELIVERYMODE	Deviation from planned delivery mode
FTP	Delayed / Failure to progress in Labour (FTP)
TPOL	Threatened premature onset of Labour (TPOL)
PLACENTAACCRETTA	Placenta Accreta (PA)
PLACENTAPREVIA	Placenta Previa (PP)
APH	Antepartum Haemorrhage (APH)
PPH	Postpartum haemorrhage (PPH)
HYPERTENSIONPIT	Pregnancy Induced Hypertension (PIH)
PET	Preeclampsia (PET)
IUGR	Intrauterine growth restriction or small for dates (IUGR)
SEPSIS	Sepsis (maternal)
**Neonatal variables that include interventions and complications**	
BABYOUTCOME1	Alive and well
BABYOUTCOME2	Neonatal Death*
BABYOUTCOME3	Intrauterine Foetal Death (IUFD)
BABYOUTCOME4	Termination of pregnancy (TOP)
BABYOUTCOME5	Prematurity <37 weeks gestation

BABYOUTCOME6	Prematurity +†NICU/SBCU3 required to stay.
BABYOUYCOME7	Active resuscitation with ‡O2 / BMV/Intubated/ IPPV
BABYOUTCOME8	Retrieval/ §CPAP/ Ventilation
BABYOUTCOME9	Active resuscitation low invasive
BABYOUTCOME10	Septic workup/ Sepsis
BABYOUTCOME11	Respiratory Distress Syndrome (RDS)
BABYOUTCOME13	Respiratory Distress Syndrome required CPAP
BABYOUTCOME12	Clinical Diagnosis of IUGR/SFD small for dates
BABYOUTCOME14	Diagnosis of congenital heart disease (CHD)
BABYOUTCOME15	Diagnosis of Congenital Abnormalities
BABYOUTCOME17	Low Apgar Score<7 at 1 minute
BABYOUTCOME18	Low Apgar score <7 at 5minutes
NICUADMISSION	NICU/SBCU3 admission
SBCU3ADMISSION	SBCU3Admission
**Maternal Cardiac variables that include required interventions, complications and outcomes**	
MATERNALOUTCOME1	Maternal Cardiac Death
MATERNALOUTCOME2	Cardiac Arrest
MATERNALOUTCOME3	Decompensated Heart Failure (HF)

MATERNALOUTCOME4	Acute Myocardial Infarction AMI
MATERNALOUTCOME5	Valvular Heart Disease (VHD) or Congenital Heart Disease (CHD) Diagnosis during pregnancy.
MATERNALOUTCOME6	Sustained Arrhythmias requiring treatment
MATERNALOUTCOME7	Supraventricular Tachycardia (SVT)
MATERNALOUTCOME8	Bradyarrhythmias without syncope or heart failure (HF)
MATERNALOUTCOME9	Non-specific chest pain
MATERNALOUTCOME10	Cardiovascular Implantable Electronic Device (CIED) i.e., Defibrillator /Permanent Pacemaker
MATERNALOUTCOME11	Cardiac Surgery required
MATERNALOUTCOME12	Balloon Valvoplasty required.
MATERNALOUTCOME13	[||]EP studies/Cardiac ablation required
MATERNALOUTCOME14	**CTPA+ Echocardiogram required
MATERNALOUTCOME15	Coronary angiogram with/without PCI required
MATERNALOUTCOME16	An episode of cardiogenic shock requiring Intra-aortic balloon pump (IABP)
MATERNALOUTCOME17	Other complications non-cardiac

Legend : *No neonatal deaths in the cohort, [†]NICU/SBCU3: neonatal intensive care unit / Special Baby Care Unit level 3, ‡ O2 / BMV/Intubated/ IPPV: oxygen therapy, bag-mask ventilation, Intermittent Positive Pressure Ventilation., [§]CPAP/ Ventilation: Continuous Positive Pressure Ventilation, [||]CTPA EP studies/cardiac ablation = electrophysiology (EP) study and cardiac ablation procedure, ** CTPA+ Echocardiogram =Computed -Tomography Pulmonary Angiogram and cardiac Echocardiogram,*** PCI percutaneous coronary intervention.
