# Supplementary material for: The association between guidelines adherence and clinical outcomes during pregnancy in a cohort of women with cardiac co-morbidities
Source: PLoS One. 2021 Jul 23;16(7):e0255070. doi: 10.1371/journal.pone.0255070 (PMC8301645; doi:10.1371/journal.pone.0255070)
Supplement: S4 Table — (PDF) [file pone.0255070.s004.pdf]

S 4 Table: Baseline Characteristics for the cohort.

Total (n=261)	Overall cohort=261	Pre-existing cardiac (PEC) n=139	Acquired cardiac (AC) n=122
Maternal Age (years)	30.4±5.8	29.7±5.6	31.2±6.0
Weight (kg)	76.4±19.5	73.7 ±17.6	79.6±21.1
Height (cm)	165±7.9	165.2±8.4	165.0±7.3
*BMI (kg/m^{2})	28±7.5	27±6.4	30.1±8.4
Born in Australia	211(80.8)	112 (80.6)	99 (81.1)
Born overseas	47 (18)	24 (17.3)	23 (18.9)
**Ethnicity**			
Aboriginal	32 (12.3)	21(15)	11 (9.0)
Asian	15 (5.7)	8 (5.8)	7 (5.7)
Caucasian	205 (78.5)	106(76)	99 (81.1)
Other	4 (3.2)		4 (3.2)
Metropolitan	201 (77)	100 (71.9)	101 (82.8)
Rural	45(17.2)	30 (21.6)	15 (12.3)
Remote	11 (4.2)	8 (5.8)	3 (2.5)

**Marital status**			
Married	160 (61.3)	78 (56)	82 (67)
De facto	54(20.7)	37 (26.6)	17 (13.9)
Divorced/ Separated	6(2.3)	2 (1.4)	4 (3.3)
Single	32(12.3)	18 (12.9)	14 (11.5)
**Perinatal assessment (SD)**			
Gestational age on admission	37.1±3.7	36.9± 4.45	36.9±3.5
†Gravida	3.1±2.08	3.0 ±1.9	3.1 ±2.2
‡Parity	1.5±1.7	1.4 ±1.6	1.5±1.8
Blood pressure **(SD)**			
○ Systolic	115±17.3	113±17.1	117±17.4
○ Diastolic	69.9±13.3	68.2±12.8	71.9±13.8
**Mental Health Scores (SD)**			
§Antenatal Risk Questionnaire, (ANRQ)	18.4±12.5	17.4±12.1	19.8±13.2
‖Edinburgh Postnatal Depression Scale (EPPDS)	6.3±5.9	6.0±5.9	6.8±5
Overall Adherence score (SD) =/40	16.7±6.5	17.7±6.4	15.2±6.2

∗BMI=Body Mass Index Kg/$m^{2,}$ † Gravida on admission: number of times a woman is or has been pregnant, regardless of the outcome. ‡Parity: number of pregnancies reaching viable gestation age (includes live births & stillbirths). §ANRQ: documented score for antenatal risk questionnaire – self-reported psychosocial assessment tool. ‖ EPPDS: documented score for the Edinburgh postnatal depression scale for risk of perinatal depression high score indicates depressive conditions.
