# Supplementary material for: The association between guidelines adherence and clinical outcomes during pregnancy in a cohort of women with cardiac co-morbidities
Source: PLoS One. 2021 Jul 23;16(7):e0255070. doi: 10.1371/journal.pone.0255070 (PMC8301645; doi:10.1371/journal.pone.0255070)
Supplement: S5 Table — (PDF) [file pone.0255070.s005.pdf]

S5 Table: Univariate linear regression model results for the adherence score versus obstetric clinical variables.

Clinical variables	Mean difference Adherence score (95% CI)	Standardized Coefficients Beta	P value	Adjusted R Square	R Square Change	n (%) N=261
**Obstetric complications.**						
Pregnancy Induced Hypertension (PIH)	3.060 (0.861,5.259)	0.168	0.007	0.024	0.028	39 (14.9)
Preeclampsia (PET)	3.202 (0.528,5.875)	0.145	0.019	0.017	0.021	25 (9.6)
Threatened premature onset of Labour (TPL)	2.857 (0.388,5.325)	0.140	0.023	0.016	0.020	30 (11.5)
Delayed / Failure to progress in labour (FTP).	0.931 (-1.636,3.497)	0.044	0.476	-.002	0.002	28 (10.7)
Placenta Accreta (PA)	2.659 (-4.794,10.111)	0.044	0.483	-0.002	0.002	3 (1.1)
Placenta Previa (PP)	4.818 (0.243,9.393)	0.128	0.039	0.013	0.016	8 (3.1)
Antepartum Haemorrhage (APH)	-1.306 (-5.660,3.049)	-0.037	0.555	-0.003	0.001	9 (3.4)
**Modes of Delivery.**						
Normal vaginal delivery	-5.358 (-6.850, -3.866)	-0.402	<0.001	0.159	0.162	104 (39.1)
Assisted vaginal delivery	-1.199 (-3.806,1.407)	-0.056	0.366	-0.001	0.003	27 (10.3)
Elective Caesarean (†LSCS)	5.316 (3.676,6.956)	0.369	<0.001	0.133	0.136	74 (28.4

Clinical variables.	Mean difference Adherence score (95% CI)	Standardized Coefficients Beta	P value	Adjusted R Square	R Square Change	N=261
Emergency LSCS	1.726 (-0.225,3.6278)	0.108	0.083	0.008	0.012	54 (20.7)
Emergency LSCS+ Hysterectomy/Laparotomy	1.569 (-4.901, 8.039)	0.030	0.633	-0.003	0.001	4 (1.5)
*Deviated from planned delivery mode.	2.000 (0.354,3.646)	0.147	0.017	0.018	0.022	92 (35.2)
**Obstetric Complications continued.**						
Postpartum haemorrhage (PPH)	0.503 (-1.802,2.808)	0.027	0.668	-0.003	0.001	36 (13.8)
Abnormal Doppler	4.413 (-0.479,9.305)	0.110	0.077	0.008	0.012	7 (2.7)
Abnormal ‡ IUGR	5.610 (1.525,9.695)	0.166	0.007	0.024	0.027	10 (3.8)
Sepsis	-3.082 (-6.478,0.313)	-0.110	0.075	0.008	0.012	15 (5.7)

Legend: Significance p value <0.2. All covariates /predictors were yes vs no. * Deviated from planned delivery mode; those women with planned NVD who required alternate listed modes or Elective LSCS deviated to precipitous SVD or Emergency LSCS. †LSCS: Lower Segment Caesarean Section. ‡ IUGR: Intrauterine Growth Restriction
