# Supplementary material for: The association between guidelines adherence and clinical outcomes during pregnancy in a cohort of women with cardiac co-morbidities
Source: PLoS One. 2021 Jul 23;16(7):e0255070. doi: 10.1371/journal.pone.0255070 (PMC8301645; doi:10.1371/journal.pone.0255070)
Supplement: S6 Table — (PDF) [file pone.0255070.s006.pdf]

S 6 Table: Univariate linear regression model results for the total adherence score versus neonatal clinical variables.

Clinical variables	Mean difference Adherence score (95% CI)	Standardized Coefficients Beta	P value	Adjusted R Square	R Square Change	n (%) N=261
*Baby Alive and well	-3.457 (-5.185, -1.729)	-0.238	<0.001	0.053	0.057	189 (72.4)
Intrauterine Foetal Death (IUFD)	2.304 (-10.564,15.171)	0.022	0.725	-0.003	0.000	1 (0.4)
Termination of pregnancy (TOP)	-6.731 (-19.575,6.114)	-0.064	0.303	0.000	0.004	1 (0.4)
**Birth complications**						
Prematurity	3.762 (1.727, 5.798)	0.221	0.001	0.045	0.049	46 (17.6)
†Prematurity+ NICU/SBCU3	3.927 (1.817,6.037)	0.222	<0.001	0.049	0.049	42 (16.1)
Active resuscitation with O2 / BMV/Intubated/ IPPV	4.093 (1.784,6.402)	0.212	<0.001	0.045	0.045	34 (13)
Retrieval/ †CPAP/ Ventilation	2.492 (-1.855,6.839)	0.070	0.260	0.001	0.005	9 (3.4)
Active resuscitation of baby (low invasive) at birth	1.698 (-0.996,4.391)	0.077	0.216	0.006	0.006	25 (9.6)
‡ Low Apgar Score<7 at 1 minute	3.092 (0.718,5.403)	0.162	0.009	0.023	0.026	35 (13.4)
Low Apgar Score<7 at 5 minutes	5.025 (1.942,8.108)	0.196	0.001	0.035	0.038	18 (6.9)
Respiratory Distress Syndrome (RDS)	4.658 (2.501,6.814)	0.256	<0.001	0.065	0.065	39 (14.9)

Respiratory Distress Syndrome (RDS) with CPAP ventilation	3.998 (1.116,6.880)	0.167	0.007	0.024	0.028	21 (8)
**Clinical variables**	**Mean difference Adherence score (95% CI)**	**Standardized Coefficients Beta**	**P value**	**Adjusted R Square**	**R Square Change**	**n (%) N=261**
[§]NICU Admission	5.240 (3.589,6.892)	0.362	<0.001	0.128	0.131	73 (28)
SBCU3 Admission	0.677 (-3.206,1.852)	-0.033	0.599	-0.003	0.001	29 (11.1)
[||]Clinical Diagnosis of IUGR/SFD.	5.194 (1.101,9.287)	0.153	0.013	0.020	0.024	10 (3.8)
Diagnosis of congenital heart disease (CHD)	6.443 (1.196,11.690)	0.149	0.016	0.018	0.022	6 (2.3)
Diagnosis of Congenital Abnormalities (other)	7.010 (2.477,11.542)	0.186	0.003	0.31	0.035	8 (3.1)
Septic workup/ Sepsis	0.854 (-2.282,3.990)	0.033	0.592	-0.003	0.001	18 (6.9)

Legend: Significance p value <0.2. All covariates /predictors were yes vs no. [*]Documented as 'Alive and Well' by clinicians at birth, [†]Prematurity required admission to neonatal intensive care unit (NICU) or special baby care unit (SBCU3). [†]Active Resus+ O_2/BMV/ intubate /IPPV: active resuscitation that required oxygen administration (O_2), bag-mask ventilation (BMV) but also required intubation (intubate) and mechanical ventilation mode of intermittent positive pressure ventilation (IPPV) CPAP: mode of ventilation Continuous Positive Pressure ventilation,[‡] Missing data totals for Apgar score<7at 1 min = 2, and at 5 min =1, [§]NICU admission baby admitted for other reasons, i.e. flat at birth, required active resuscitation, RDS, hypothermia, for observations and as boarder due to maternal issues, [||] IUGR/SFD intrauterine growth restriction/ small for dates.
