# Supplementary material for: The association between guidelines adherence and clinical outcomes during pregnancy in a cohort of women with cardiac co-morbidities
Source: PLoS One. 2021 Jul 23;16(7):e0255070. doi: 10.1371/journal.pone.0255070 (PMC8301645; doi:10.1371/journal.pone.0255070)
Supplement: S7 Table — (PDF) [file pone.0255070.s007.pdf]

S7 Table:. Univariate linear regression model results for the adherence score versus maternal cardiac clinical variables.

**Clinical variables**	**Mean difference Adherence score (95% CI)**	**Standardized Coefficients Beta**	**P value**	**Adjusted R Square**	**R Square Change**	**n (%) N=261**
Acquired cardiac group	-2.278 (-3.847, -0.709)	-0.175	0.005	0.027	0.031	122 (46.7)
Pre-existent cardiac group	2.278 (0.709, 3.847)	0.175	0.005	0.027	0.031	139 (53.2)
**Complications and clinical outcomes.**						
Maternal Cardiac Death.	-9.742 (-22.558, 3.073)	-0.093	0.136	0.005	0.009	1 (0.4)
Cardiac Arrest.	2.016 (-1.933,5.966)	0.062	0.316	-0.000	0.004	11 (4.2)
Decompensated Heart failure.	4.353 (1.925,6.145)	0.246	<0.001	0.061	0.061	42 (16.1)
Acute Myocardial Infarction (AMI).	-4.185 (-9.963,1.593)	-0.088	0.155	0.004	0.008	5 (1.9)
Valvular Heart Disease (VHD) or Congenital Heart Disease (CHD) New Diagnosis.	0.410 (-1.448,2.267)	0.027	0.664	-0.001	0.001	63 (24.1)
Sustained Arrhythmias requiring treatment.	-0.055 (-1.792,1.682)	-0.004	0.951	-0.004	0.000	78 (29.9)
Supraventricular Tachycardia (SVT).	-0.256 (-2.027,1.515)	-0.018	0.776	-0.004	0.000	73 (28)
Bradyarrhythmias without syncope or heart failure (HF).	-1.001 (-4.796, 2.794)	-0.032	0.604	-0.003	0.001	12 (4.6)
Non-specific chest pain.	0.584 (-1.405,2.574)	0.036	0.563	-0.003	0.000	52 (19.9)

Clinical variables.	Mean difference Adherence score (95% CI)	Standardized Coefficients Beta	P Value	Adjusted R Square	R Square Change	n (%) N=261
**Cardiac investigations during pregnancy included diagnostic and treatment.**						
Coronary angiogram with and without *PCI.	-1.349(-4.874,2.177)	-0.047	0.452	-0.002	0.002	14 (5.4)
†CTPA+ Echocardiogram.	-2.064 (-4.707,0.578)	-0.095	0.125	0.009	0.009	26 (10)
‡EP studies/Cardiac ablation.	-1.766 (-6.118, 2.587)	-0.050	0.425	-0.002	0.002	9 (3.4)
Cardiovascular Implanted Electrical Device (CIED)	4.396 (-0.882,9.674)	0.101	0.102	0.006	0.010	6 (2.3)
Cardiac Surgery required.	8.149 (2.938,13.360)	0.188	0.002	0.032	0.035	6 (2.3)
Balloon Valvoplasty procedure during pregnancy.	9.403 (2.033,16.773)	0.154	0.013	0.020	0.024	3 (1.1)
Cardiogenic Shock with intra-aortic balloon pump (IABP).	1.300 (-11.570,14.170)	0.012	0.842	-0.004	0.000	1 (0.4)
**Other non-cardiac complications**						
‡Other non-cardiac complications	2.863 (-0.437,6.163)	0.106	0.089	0.007	0.011	16 (6.2)

Legend: Significance p value <0.2. All clinical variables were yes vs no. * PCI =percutaneous coronary intervention, CTPA+ Echocardiogram= Computed -Tomography Pulmonary Angiogram and cardiac echocardiogram ‡EP studies/Cardiac ablation = electrophysiology (EP) study and cardiac ablation procedure. §Other non-cardiac complications included ruptured splenic artery –laparotomy and splenectomy, hepatic encephalopathy and bleeding gastric varices, oesophageal sclerotherapy.
