# Supplementary material for: The association between guidelines adherence and clinical outcomes during pregnancy in a cohort of women with cardiac co-morbidities
Source: PLoS One. 2021 Jul 23;16(7):e0255070. doi: 10.1371/journal.pone.0255070 (PMC8301645; doi:10.1371/journal.pone.0255070)
Supplement: S8 Table — (PDF) [file pone.0255070.s008.pdf]

S 8 Table: Cardiac arrest cases, associated pathology and outcomes.

Timing	Pathology	Outcomes
First trimester antepartum	Primigravida with no comorbidities. Out of hospital cardiac arrest of Ventricular Fibrillation (VF) with paramedics in attendance.	Resuscitation with hospital admission for intensive care therapy and cardiovascular implantable electronic device (CIED). Live baby at term.
Second trimester antepartum	Multigravida with comorbidities of Rheumatic heart Disease (RHD), Mitral regurgitation (MR). Cardiac arrest with current pregnancy complicated by pneumonia, severe preeclampsia and eclampsia seizure.	The mother was resuscitated, treated with hydralazine, magnesium sulphate with early emergency LSCS. Baby and mother survived and followed up by cardiology.
Second trimester antepartum	Multigravida presented to the hospital with evidence of intracranial bleed (unknown aetiology), cardiac arrest with sudden Acute Pulmonary Oedema (APO).	Intensivist referred to the cardiologist—multidisciplinary team discussion with retrieval to a higher care facility. Mother resuscitated with advanced life support in transit, required neurosurgery and emergency LSCS—diagnosis of ongoing left ventricular failure, provisional peripartum cardiomyopathy. Preterm newborn required active resuscitation and admission to the neonatal intensive care unit (NICU).
Second trimester antepartum	Multigravida with comorbidities of chronic hypertension and Gestational Diabetes (GD). Hospital admission for chest pain and dysponea rapidly deteriorated with APO and cardiopulmonary arrest.	She was resuscitated with advanced life support. Hypertension treated with IV hydralazine, esmolol, lignocaine 1% 80mg, and magnesium sulphate infusion preceded emergency LSCS and subsequent retrieval to a higher care facility for intensive care therapy diagnosis of preeclampsia with APO and diastolic heart failure. Medical follow up for chronic hypertension. The baby required resuscitation at birth and admission to NICU for prematurity
Second trimester postpartum	Multigravida, with history of Rheumatic Fever (MR), Polycystic ovary syndrome, cholecystectomy. This pregnancy precipitous preterm delivery of the first baby of multiple births *at home,* subsequent deliveries in hospital complicated by unconscious maternal collapse postpartum.	The mother survived in the postnatal ward with the resuscitation team in attendance and followed up with cardiology. Triplets required admission to NICU for prematurity.
Second trimester Antepartum	Multigravida, with known mixed valve disease RHD, left brachial artery thrombosis. Recurrent tracheal papillomas and diagnosis of granuloma gravidarium of the trachea accompanied by severe mitral regurgitation, eccentric left	Mother and baby survived with advanced life support measures by the retrieval team—emergency LSCS following retrieval to the higher obstetric care facility. The preterm baby required active resuscitation, respiratory support in NICU for 48 hours.

S 8 Table: Cardiac arrest cases, associated pathology and outcomes.

	ventricular hypertrophy. Respiratory followed by cardiac arrest.	Cardiothoracic surgery with cardiopulmonary support occurred 24 hours post-delivery for debulking of the tumour.
Third trimester antepartum	Primigravida. Pregnancy complications of gestational diabetes and chronic hypertension. Rapid deterioration whilst attending the blood pressure monitoring clinic with cardiac arrest, ECG: Ventricular Tachycardia (VT) Acute Pulmonary Oedema (APO), (*Post cardiac arrest undeclared congenital condition previous atrial switch procedure).*	Protracted resuscitation with advanced life support and emergency LSCS. Retrieval to a higher care facility for intensive care treatment for subsequent cardiogenic shock and long term rehabilitation. Baby retrieved to NICU. Both mother and baby survived, with long term cardiology follow-up.
Third trimester at delivery	Primigravida, a high risk pregnancy complicated by antepartum haemorrhage (APH) treated with blood transfusion. Hospitalised when placenta praevia grade IV and accreta were noted. Emergency LSCS for APH and hysterectomy. Cardiac arrest occurred during the anaesthetic preparation, with VT.	Resuscitation managed by an anaesthetic team with CPR and Iv drugs whilst the surgeon closed the abdominal wound, having achieved haemostasis. Normalisation of the ECG (Sinus tachycardia with ST-segment depression) and Cardiac output. The woman required respiratory support and intensive care treatment for a further 24 hours.
Third trimester at delivery	Multigravida planned (repeat) LSCS for breech presentation (*no issues with spinal*). Cardiac arrest with Asystole on ECG during delivery.	She was resuscitated with CPR, IV drugs and urgent LSCS for foetal bradycardia. Mother and baby alive and well. Post cardiac arrest anaesthetic and cardiology follow up.
Third trimester at delivery	Primigravida, comorbidities of familial hyperlipidaemia and an incidental finding of Wolf Parkinson White syndrome (WPW) on ECG following cardiology review. Pregnancy complications of preeclampsia and pulseless electrical activity (PEA) cardiopulmonary arrest, post epidural analgesia during labour.	Resuscitated with 20 minutes of CPR and IV drugs, until ECG, and cardiac output (CO) restored. The woman required invasive respiratory support, therapeutic hypothermia, and intensive care therapy for 48 hours—mother followed up by cardiology. Finally, the baby boarded in NICU.
Third trimester postpartum	Multigravida comorbidities of chronic hypertension, gestational diabetes, recent diagnosis of left bundle branch block (LBBB) on ECG. Unwitnessed VF cardiac arrest while an inpatient (unmonitored).	Resuscitation protracted with advanced life support and ongoing ICU management for respiratory support, further cardiac investigations, and CIED. Finally, the mother required residential care and long term respiratory support following complication of global hypoxic encephalopathy.
