# Supplementary material for: The association between guidelines adherence and clinical outcomes during pregnancy in a cohort of women with cardiac co-morbidities
Source: PLoS One. 2021 Jul 23;16(7):e0255070. doi: 10.1371/journal.pone.0255070 (PMC8301645; doi:10.1371/journal.pone.0255070)
Supplement: S9 Table — (PDF) [file pone.0255070.s009.pdf]

S 9 Table: Cardiac, obstetric, and other indications for the Emergency /Elective lower segment caesarean sections.

Cardiac Indications ± Obstetric complications	Frequency	Women=Deviation from plan
Valve disease with preeclampsia.	n=2	n=2
Valve disease with decompensated heart failure.	n=3	n=3
Stable severe stenotic valve disease.	n=1	n=1
Dilated cardiomyopathy resulting in severe pulmonary hypertension and / or decompensated heart failure.	n=5	n=5
Worsening primary pulmonary hypertension with heart failure.	n=1	n=1
Marfans Syndrome, with significant aortic root dilation.	n=3	n=2
Type B aortic dissection antepartum.	n=1	n=1
Complex Cardiovascular Anatomy with preeclampsia.	n=1	n=1
Cardiac arrests.	n=3	n=3
Acute pulmonary oedema of unclear cause.	n=1	n=1
**Obstetric Indications**		
**Antepartum:**		
Placental Abruption (n=1)/ Placenta Accreta /Placenta Previa, Grade III and IV /Antepartum haemorrhage.	n=16	n=1
Pregnancy-induced hypertension (23)/ Preeclampsia and eclampsia (25) with additional obstetric complications.	n=48	n=25

Obstetric Indications continued	Frequency	Women= deviation from plan
**Delivery related**:		
Repeat Lower Segment Caesarean Section (LSCS) +	n= 70	n=50
Failed to progress (FTP)/instrumental delivery or induction of labour + Cord prolapse (n=1).	n=27	n=27
Malpresentation: Breech / Brow + cholestasis of pregnancy.	n= 6	n=6
Threatened premature onset labour and /premature onset of labour, with MCDA twins and malpresentation.	n=19	n=19
Failed instrumental delivery or induction of labour (IOL).	n=5	n=5
Premature rupture of membranes <24 hours with additional obstetric complications, e.g., threatened premature labour (TPL) with a congenital cardiac condition.	n=6	n=5
**Obstetric Baby related:**		
Foetal distress, e.g., Foetal Tachycardia /bradycardia.	n=13	n=13
Abnormal Doppler (n= 5) /‡IUGR/(N=6) /Asymmetrical growth.	n= 11	n=11
IUGR + Polyhydramnios/ Oligohydramnios low liquor.	n=5	n=5
Multiple births, i.e., §MCDA/ DCDA twins with growth discrepancy, malpresentation or past LSCS.	n=3	n=3
**Other indications**		
Maternal Myotonic Muscular dystrophy (MMD) plus uterine dehiscence.	(n=1)	n=1
‖. IVF pregnancy + maternal request, history of pregnancy loss, FTP, repeat LSCS (n=2) + hypertensive heart disease.	(n=3)	n=1
Preexisting poorly controlled hypertension with decompensated heart failure.	(n=1)	n=1

Legend, ‡IUGR: intrauterine growth restriction, §MCDA: monochorionic diamniotic twins share the same chorionic sac but have two amniotic sacs, DCDA: dichorionic diamniotic twins each twin has its own chorionic and amniotic sac, ‖IVF: in vitro fertilisation. NB: There were women with multiple adverse clinical factors for obstetric indications for LSCS; therefore, overlapping of indications occurred.
