# Supplementary material for: The association between guidelines adherence and clinical outcomes during pregnancy in a cohort of women with cardiac co-morbidities
Source: PLoS One. 2021 Jul 23;16(7):e0255070. doi: 10.1371/journal.pone.0255070 (PMC8301645; doi:10.1371/journal.pone.0255070)
Supplement: Attachment — Submitted filename: Tabulated Rsponse to Reviewers 21062021.docx [file pone.0255070.s010.docx]

| **Reviewer #1 feedback comments** | Author’s comment | Response | Page Number  (Clean copy) |
| --- | --- | --- | --- |
| Has the statistical analysis been performed appropriately and rigorously? | All statistics were performed under the supervision of a medical statistician who is also a co-author (Suzanne Edwards). The description of the model building completed in this paper is considered to be appropriate and rigorous based on Heinze and Dunkler 2017 |  |  |
| To understand this article better, it is necessary to thoroughly review the study where the authors evaluated adherence to the guidelines. However, It could be helpful to add a brief description of the design aspects and the previous publication to permit the readers to inquire about the topic. | We have included a summary of our previous findings in the paper's introduction. We have not re-iterated the methods section of our last article as this is repetitive but referred to it. Therefore, like papers that have previously published their protocol and do not repeat the same information in the results paper. We hope this is satisfactory | In summary, we showed that variance in adherence correlated with the exposure to the higher acuity cases and appropriate up-referral to the only high risk referral centre for the state, the quartenary hospital two, which provided (level six) care, i.e. maternal cardiac and neonatal intensive care services. Likewise, hospital one offered intermediate care (level five) inclusive of tertiary maternal services, intensive care and specialised neonatal care services but excluded babies less than 32 weeks. Also, hospital three was a stand-alone maternity unit attached to a large neonatal intensive care, yet without a maternity intensive care service onsite (20).  The previous study evaluated the adherence to recommendations within the guidelines. The predictors for adherence were examined, such as hospital sites, the two categories of cardiac disease, underlying cardiac causes, risk level of pregnancy, multidisciplinary team collaboration, and maternal and baby demographic factors. The participants included only women with 'preexistent' and 'newly acquired' heart disease during pregnancy, with a more comprehensive discussion on the selection criteria presented in Millington (2020) et al. (20).  As reported in the previous study, adherence to guidelines was calculated with a purposefully designed scoring system. Scoring encompassed preconception counselling, antenatal care (clinical assessment and investigations), foetal wellbeing assessments, planned risk stratification, mode of delivery and labour recommendations as per guidelines (20). | Page 6-7, lines 121 -134.  .  Page 7, Lines 135-138 |
| I consider it is essential to highlight the difference between the three hospitals mentioned | The authors thank the reviewer for comments regarding the difference between the three hospitals.  The authors agree that the difference between the three hospitals is important and was described in the previous publication. But this paper singular narrative aims to describe the frequencies of clinical variables, i.e. morbidities, outcomes and interventions inclusive of the cardiac groups of the women during pregnancy and their association with adherence scores. Therefore, the three hospitals were not included in the analysis for the following reasons.   - The three tertiary hospitals identified in the study were in the same metropolitan city with access to the same recommended statewide perinatal guidelines for practice. - The differences in real-life practice between the three hospitals were addressed in the 2020 publication; please refer to Millington SK et al. 2020 (20). The findings showed that across three SA Health public metropolitan, university-affiliated hospitals, the variance in adherence across the three hospitals correlated with the exposure to higher acuity cases. - As outlined in the same publication, there was cooperation between the three sites for interhospital transfer in high-risk cases. - Given the number of women in the cohort and the fact that the numbers were *not equally matched* for each hospital site, reducing the power to analyse, it was more feasible to look at the overall score for adherence in linear regression modelling and cardiac groups. - The disparities between the hospitals were not the aim of this paper and were highlighted in the previous publication. |  | Refer to Page 6-7, lines 121 -134. |
| Although maternal mortality was very low, the number of patients who presented with cardiac arrest was significant, so it would be relevant to report which were the pathologies that were associated with cardiac arrest in the description of outcomes (it could be a table) | The authors thank the reviewer for requesting the description of outcomes and associated pathologies with the eleven cardiac arrest cases presented in Table VIII. This may be appropriate to include as a supplementary. | Table VIII. Cardiac arrest cases, associated pathologies and outcomes. |  |
| Caesarean section deliveries were very high. It would be interesting to know the indication for caesarean section (cardiac vs obstetric) since most heart diseases can end by vaginal delivery, leaving a cesarean section for a cardiac indication to very selected cases. It would be essential to know more characteristics that help define why the high rate of caesarean sections. The authors mention that state practices do not suggest elective caesarean section as the first option (and neither do the other pregnancy and heart disease guidelines). They recommend that this enhanced the results for both: mother and baby. So, it is essential to know in which scenarios the cesarean section was performed because if it was in severe and unstable conditions, it would be justified. | Although the reviewer is concerned about the high percentage of LCSC rate in our cohort, the Australian Institute of Health and Welfare indicates that the LCSC rate for women giving birth for the first time from 2004 to 2018 in South Australia was 29.5% and overall in Australia was 30.1%. (<https://www.aihw.gov.au/reports/per/095/ncmi-data-visualisations/contents/labour-birth/b5>).  The elective LCSC rate in our cohort of 261 was n= 74, 28.4%; however emergency LCSC rate was n=54, (20.7 %,) making our LCSC rate 49.1%. Solely cardiac indications for LCSC occurred in 20 women (7.6%), whereas obstetric or combined obstetric and cardiac indications occurred in the others. We found that the rationale for LCSC was frequently complex, and the decision was not based on a single parameter.  This level of clinical complexity has been noted by previous registries (CAREPREG and ROPAC).  In the CAREPREG study cohort of 546 women who completed pregnancies, 98% were live births, with 164 deliveries by caesarean section Most caesarean deliveries (96%) were for obstetric indications; maternal cardiac status was indicated in 4% as reported in Siu et al. 2001.  The ROPAC study with a focus on structural heart disease described two cohorts :  **PREG 1** cohort of 1,321 data collected before 2011 with reported LSCS 2,681 (46.7%) and emergency LSCS 766 (13.3%).  **PREG 2** cohort of 5,739 data collected from 2011 onwards with reported LSCS 2143(48.6%) and emergency LSCS 562 (12.7%).  Finally, in our cohort, the cardiac scenarios resulting in a decision for elective or emergency LCSC were *(n=20) and those women who deviated from the delivery plan ( n= 13 )*:  Valve disease with preeclampsia n=2  Valve disease with decompensated heart failure n=3  Stable but severe stenotic valve disease n=1  Dilated cardiomyopathy resulting is severe pulmonary hypertension and/or decompensated heart failure  n=5  Worsening primary pulmonary hypertension with heart failure  n=1  Marfans’ syndrome with significant aortic root dilatation  n=3  Type B aortic dissection antepartum n=1  Complex congenital heart disease with preeclampsia  n=1  cardiac arrest n=3  acute pulmonary oedema of unclear cause n=1  In general, the deviation of the plan from NVD to LCSC was related to a change in the clinical state with haemodynamic compromise or a new serious complication (cardiac, obstetric, neonatal or other medical reasons).  We have categorised the indications for LCSC in Table IX, which may be an appropriate supplementary table. | Table IX Indications for Emergency and elective LSCS |  |
| 30-40% of patients had deviation in the delivery plan. As far as possible, given the retrospective nature, it would be good to know why the change occurred (change in the clinical condition that caused an indicated change or preference of the treating physician), as this has implications for quality of care and the impact of the concept of the multidisciplinary group. | We have included the number and reasons for deviation from the planned NVD to LCSC in table IX, so this should answer the reviewer's query. |  |  |
| The acronyms should be reviewed as some do not correspond or are not referenced before their use. | The authors thank the reviewers for the feedback on acronyms. The manuscript has been checked for inconsistencies with abbreviations where they are not referenced before their use.   1. Cardiovascular disease( CVD ) 2. lower segment caesarean sections (LSCS ) 3. the Cardiac disease in Pregnancy Study (CAREPREG II) 4. ECMO 5. Cardiovascular Implantable Electrical Device (CIED) Table 1 | 1. During pregnancy, cardiovascular disease (CVD) management guidelines are informed by knowledge generated through case reports, registries, and expert opinion rather than evidence from randomised clinical trials (2, 5, 21). 2. (forceps and vacuum device), both elective and emergency lower segment caesarean sections 3. This cohort reflected similar findings to the Cardiac disease in Pregnancy Study (CAREPREG II) study that cardiac complications in pregnant women with heart disease are primarily related to maternal arrhythmias and heart failure (Table 1) (28). 4. Two examples include intracranial bleeding treated with craniotomy and tumour in the distal trachea treated post-delivery with extracorporeal membrane oxygenation (ECMO) support thoracotomy and debulking of tumour and insertion of a tracheal stent. 5. Table 1 legends have been updated where LSCS and CIED to ensure full reference included, and so track changes will be included as well as revised supplementary tables | Page 5 lines 93  Page 10 lines 218  Page 26, line 368.  Page 31 Lines 488. |
| **Reviewer #2 comments** |  |  |  |
| Multiple clinical outcomes were presented: Cardiac variables, obstetric variables and neonatal variables. Specific questions include: |  |  |  |
| Was there data on congenital neonatal conditions, in particular neonatal heart disease, and how did that relate to the outcomes? | The authors thank the reviewer for their feedback on congenital neonatal conditions, particularly neonatal heart disease, and outcomes. The primary and secondary outcomes of neonatal mortality and morbidity are discussed earlier in the manuscript. Please refer to page 10.  "Preexistent maternal heart disease, possibly via a decline in maternal cardiac output during pregnancy (IUGR) and obstetric complications, are associated with an increased risk of neonatal complications (14, 19). Therefore, this study collected data for neonatal complications…."  A small number (n=6) of neonates with congenital conditions diagnosed at birth or post-delivery did not influence overall adherence. All the babies survived, with some requiring further paediatric care and follow up, but the only neonatal variable with statistically significant association with guideline adherence score was NICU admission (P <0.001). Therefore, this data includes a small number of babies (n=6) diagnosed with congenital heart disease and abnormalities. Please refer to supplementary table S II on the neonatal diagnosis of congenital heart disease and abnormalities captured in the cohort with maternal cardiac status. The timing of the babies diagnosis occurred is made explicit for readers*.* | Supplementary Table S II Neonatal diagnosis of congenital heart disease and abnormalities |  |
| What was included in the multivariate model? | The authors thank the reviewers for feedback on what variables were included in the multivariable model. The manuscript outlined the statistical process in the manuscript with both univariate supplementary tables for transparency of the process see tables SV-SVII. In addition, the initial multivariable regression analysis for the separate cardiac, obstetric, and neonatal models has been presented in manuscript results section Tables 2-4. However, the manuscript has now been revised to improve clarity. | The authors performed multivariable regression analysis using a p value cut-off criterion of 0.2 on the univariate linear regression results (38, 39). Specifically, the inclusion of all covariates with p value <0.2 on univariate regression analysis was applied in an initial multivariable model, followed by backward elimination until all remaining covariates had a p value < 0.2 (39) refer to Tables SV-VII. Multivariable regression modelling with backwards elimination was undertaken on three separate regression models for obstetrics, neonatal and cardiac predictors, respectively (refer to tables 2-4). This is how the final multivariable parsimonious model (presented in Table 5) was built. | Page 11-12, lines 243- 250. |
| Was there a power analysis done for the outcomes of interest, many were rare events | The authors thank the reviewers for feedback on the power analysis for outcomes of interest, given that many were rare events. The sample size calculation and power calculation was performed and reported in a previous publication based on adherence to guidelines, which is the primary research topic (20). Therefore sample size and power calculations are based on the primary hypothesis as reported in the previous paper. Clinical outcomes are a secondary topic. | A sample size and power calculation were performed and reported in a previous paper on this topic (20) based on adherence to guidelines. The sample size obtained provides a substantial 'margin for error' to compensate for missing data, but possibly not for rare events such as maternal deaths (n=1). | Page 9, lines 187-190. |
| Most of the presentation of results seems to be a comparison of the outcomes in the preexistent versus | The authors thank the reviewers for comment regarding the presentation of results. The two cardiac groups were not directly compared in this study. Still, they were included as binary variables in the cardiac complications /interventions regression model, where the outcome was the total adherence score. The comparison of total adherence score in the preexistent cardiac group was found to be significant ( p = 0.004 ) Table 5 |  |  |
| **Reviewer #3 comments** |  |  |  |
| In Discussion is emphasised that the association between the adherence score to the guidelines and several clinical variables indicate improved outcomes with greater adherence (line 315), but this statement must be better argued. It is implied that the outcomes are improved with greater GL adherence because the group of newly diagnosed women has a lower adherence to the guidelines and worse outcomes, but it needs to be explained. | The authors thank the reviewers for the feedback on the opening statement in the discussion section, referenced with Table 2, where the final multivariable linear regression model results are presented. Accordingly, the manuscript revised to reflect the findings and statement.  This comprehensive retrospective study supports the hypothesis of an association between the adherence score to the guidelines and some clinical variables indicating improved outcomes with greater adherence (Table 2). But the most significant was decompensated heart failure (p < 0.001) and elective LSCS elective (p <0.001) contingent upon the inclusion of multidisciplinary team collaboration in high risk pregnancies, therefore, increased adherence as previously reported (20).  Further discussion is included in the manuscript on heart failure and delivery modes pp 29-30. | This comprehensive retrospective study supports the hypothesis of an association between the adherence score to the guidelines and some clinical variables indicating improved outcomes with greater adherence (Table 2). But the most significant was decompensated heart failure (p < 0.001) and elective LSCS elective (p <0.001), contingent upon the inclusion of multidisciplinary team collaboration in high risk pregnancies (20).    **Modes of delivery**  This association demonstrates a consensus on the obstetric rationales for LSCS in women with cardiac conditions, medical conditions, and prior pregnancy complications who are more likely to experience obstetric and neonatal complications (14-19).  Women identified with adverse pregnancy outcomes such as malpresentation, premature onset of labour, multiple births, or clinical deterioration prompted elective LSCS to ensure the best result for mother and baby. As expected, the cardiac scenarios resulting in a decision for emergency or elective solely cardiac indications were small (n=20, 7.6%). Likewise, the rationale for elective LSCS included common obstetrical complications such as repeat LSCS (28%) placental dysfunction, i.e., placenta praevia and accreta (6%), pregnancy-induced hypertension preeclampsia, and eclampsia (18%), delivery related, i.e. fail to progress (10%), malpresentation (2%) and baby-related such compromised foetal wellbeing (4.9%)  Often multiple adverse pregnancy complications influenced clinicians' decision to proceed with surgical option of delivery.  Early inclusion of multidisciplinary team for surgical delivery options facilitated the uptake of guidelines recommendations such as assessing cardiac status. | Pages 26, lines 370-374.  Page29, Lines 442-478.  . |
| Since to be aware of the presence of heart disease and follow GL antenatal care is critical to evitate worse complications (i.e., maternal cardiac death, severe morbidity after cardiac arrest, Discussion line 319-346), is there any parameter in the score that can increase the sensitivity of the diagnosis of heart disease in the first trimester? Can you specify if are there GL indications on routine CV screening and if are considered in your score? (Discussion lines 359-369) | We thank the reviewers for the feedback question on which parameters in the score are more likely to increase sensitivity to heart disease diagnosis in the first trimester?  No single parameter increases sensitivity to diagnosis of heart disease; however, clinicians' awareness and preparedness of women's preexisting cardiac conditions and the guideline appropriate care helps anticipate potential pregnancy complications. Therefore clinical physical assessment and accurate history taking in the first trimester are strongly recommended. Likewise, in women with acquired cardiac conditions, the health care professional's clinical intuitiveness to adjust the care trajectory as per the guidelines may mitigate severe complications—discussed in manuscript page 28.  The clinical audit examined routine CV screening in the overall adherence score, the healthcare professional who assessed the woman at the first visit. Still, the healthcare professional's expertise and knowledge who assessed the pregnant women at the first antenatal visit influenced the information captured.  Routine prenatal health (cardiac) screening is not undertaken on all pregnant women. In South Australia over the last twenty years, midwives triage booking visits, which has worked well for most women and has improved detection of psychosocial issues and domestic violence. But there is no medical input. Therefore depending on the expertise and knowledge of midwives of risk factors, i.e. IV drug user, indigenous and RHD, cardiac /family history might be missed.  Importantly, the perinatal guidelines for women with cardiac disease in pregnancy recommend identifying women with previous pregnancies and cardiac history, cardiac assessment, preconception cardiac education where applicable, i.e. preexistent cardiac group and specific cardiac investigations and neonatal risk assessment pertinent to cardiac conditions. This information was collected in the clinical audit, scored and reported in the logistic regression analysis in Millington et al. (2020). | The authors recommend that skilled medical staff review the cardiovascular status of pregnant women with associated risk factors, such as suspected RHD in the indigenous population, family cardiac history and known illicit drug user. | Page 28, lines 427-429. |
| Discussion lines 389-397 needs to be clarified: The elective LSCS mode of delivery showed a statistically significant association with increased adherence to the guidelines. The rationale for elective LSCS included common obstetrical indications but it prompted early admission to hospital for cardiac optimisation. In our cohort, the high rate of elective LSCS (although not considered the first option in the statewide guidelines) but with increased guideline adherence scores related to other aspects of the guidelines yet improved the overall clinical outcome for both mother and baby. This result supports the safety of elective LSCS in severe and unstable cardiac conditions. What unstable cardiac conditions? What kind of cardiac optimisation is done? | The information in Supplementary Table S IX should help to clarify the reviewer's concerns.  Cardiac optimisation included advanced life support in cardiac arrest, diuresis in decompensated heart failure, respiratory support to manage hypoxia, inotropic support to manage cardiogenic shock, haemodynamic monitoring during and immediately after delivery and management of cardiac arrhythmias with anti-arrhythmic medication, DC cardioversion or implantation of an internal cardiac electrical device. We have included this in the discussion to assist in the clarification as requested. | Supplementary Table IX Indications for Emergency and elective LSCS  This included advanced life support in cardiac arrest, diuresis in decompensated heart failure, and respiratory support to manage hypoxia. Likewise, women required inotropic support for cardiogenic shock, haemodynamic monitoring during and immediately after delivery and management of cardiac arrhythmias with anti-arrhythmic medication, DC cardioversion or implantation of an internal cardiac electrical device. | Page 30, lines 446-471. |
| In Methods (line131-134) needs to be clarified how is calculated "the total adherence score", unless reporting table of the previous study, at least by mentioning the criteria by which it is computed. Moreover, the sentence "The researchers agreed on the minimum acceptable score of 17 for the newly acquired cardiac group and 35 for the preexistent cardiac group". Needs to be better explained | The authors thank the reviewers for further clarification on calculating the total adherence score and minimum acceptable score for the two cardiac groups. Calculation of total score was reported in earlier publication and achieved by quantifying perinatal guidelines, i.e. planned care variables were used to calculate the total score of 40 (N=40). The manuscript has been revised as requested. | Scoring encompassed preconception counselling, antenatal care (clinical assessment and investigations), foetal wellbeing assessments, planned risk stratification, mode of delivery and labour recommendations as per guidelines (20). A scoring system was devised that measured adherence variables used in this study were equally weighted, giving a maximum score of forty to measure adherence. Positive documentation of the guidelines, regardless of the entry point, achieved a score  A minimum score of acceptable guideline adherence was determined after comparing the two cardiac groups mean, and median adherence scores with expert review of selected cases identifying minimum expected care. From this analysis, a score of 35 (for 'preexistent') and 17 (for 'newly acquired') cardiac conditions were deemed acceptable guideline adherence. | Page 7, lines 142 – 151. |
| In results when it comes to mean guideline adherence score for groups of patients is not clear how it is calculated (e.g. at lines 284-285: Women with HF had a mean guideline adherence score of 3.546 units higher than those who did not (mean difference =3.546, 95% CI, (1.689, 5.403)). | The authors thank the reviewers for further clarification on guideline adherence score for groups of patients is calculated. The final Multivariable linear regression results Table 2 includes the unstandardised coefficient beta, and so the mean difference is the calculation from the unstandardised beta coefficient linear regression equation. | Women with HF had a mean guideline adherence score 3.546 units higher than those who did not have heart failure (mean difference (unstandardised beta coefficient from linear regression equation) =3.546, 95% CI, (1.689, 5.403). | Page 19 Lines 317 – 319. |

Heinze, G., & Dunkler, D. (2017). Five myths about variable selection. *Transplant International, 30*(1), 6-10. doi:<https://doi.org/10.1111/tri.12895>

Millington, S., Arstall, M., Dekker, G., Magarey, J., & Clark, R. (2020). Adherence to clinical practice guidelines for South Australian pregnant women with cardiac conditions between 2003 and 2013. *PLoS One, 15*(3), e0230459-e0230459. doi:10.1371/journal.pone.0230459.

Millington S, Magarey J, Dekker GA, Clark RA. Cardiac conditions in pregnancy and the role of midwives: A discussion paper. Nursing Open. 2019 0(July 23). doi: 10.1002/nop2.269.

Siu, S. C., Marcotte, F., Taylor, D. A., Gordon, E. P., Spears, J. C., Tam, J. W., . . . Investigators, C. (2001). Prospective multicenter study of pregnancy outcomes in women with heart disease. *CIRCULATION, 104*(5), 515-521. doi:10.1161/hc3001.09343.

Silversides, C. K., Grewal, J., Mason, J., Sermer, M., Kiess, M., Rychel, V. . . . Siu, S. C. (2018). Pregnancy Outcomes in Women With Heart Disease: The CARPREG II Study. *Journal of the American College of Cardiology, 71*(21), 2419-2430. <https://doi.org/10.1016/j.jacc.2018.02.076>.

Roos-Hesselink, J., Baris, L., Johnson, M., De Backer, J., Otto, C., Marelli, A. . . . Hall, R. (2019). Pregnancy outcomes in women with cardiovascular disease: evolving trends over 10 years in the ESC Registry of Pregnancy and Cardiac disease (ROPAC). *European Heart Journal, 00*, 1-8. doi:10.1093/eurheartj/ehz136.
